# Stepped-care versus treatment as usual in panic disorder: A randomized controlled trial

**DOI:** 10.1371/journal.pone.0237061

**Published:** 2020-08-13

**Authors:** Mirjam Kampman, Anton J. L. M. van Balkom, Theo Broekman, Marc Verbraak, Gert-Jan Hendriks

**Affiliations:** 1 Overwaal, Centre of Expertise for Anxiety Disorders, OCD, and PTSD part of Institute for Integrated Mental Health Care “Pro Persona”, Nijmegen, The Netherlands; 2 Behavioural Science Institute, Radboud University, Nijmegen, The Netherlands; 3 Amsterdam UMC, Psychiatry, Amsterdam Public Health Research Institute and GGZ inGeest, Specialized Mental Health Care, Vrije Universiteit, Amsterdam, The Netherlands; 4 Bureau Bêta, Nijmegen, The Netherlands; 5 HSK Groep, Arnhem, The Netherlands; 6 Department of Psychiatry, Radboud University Medical Centre Nijmegen, Nijmegen, The Netherlands; Brown University, UNITED STATES

## Abstract

Stepped-care (SC) models for anxiety disorders are implemented on a large scale and are assumed to be as effective for the greater majority of patients as more intensive treatment schemes. To compare the outcomes of SC and international guideline-based treatment (Treatment as Usual: TAU) for panic disorder, a total of 128 patients were randomized to either SC or TAU (ratio 2: 1, respectively) using a computer generated algorithm. They were treated in four mental health care centres in the Netherlands after therapists had been trained in SC by a senior expert therapist. SC comprised 10-week guided self-help (pen-and-paper version) followed, if indicated, by 13-week manualized face-to-face cognitive behavioural therapy (CBT), with medication- if prescribed- kept constant. TAU consisted of 23-week regular face-to-face CBT (RCBT) with medication -when prescribed- also kept constant. The means of the attended sessions in the SC condition was 5.9 (SD = 4.8) for ITT and 9.6 (SD = 9.6) for the RCBT condition. The difference in the number of attended sessions between the conditions was significant (*t*(126) = -3.87, *p* < .001). Remission rates between treatment conditions did not differ significantly (SC: 44.5%; RCBT: 53.3%) and symptom reduction was similar. Stepping up SC treatment to face-to-face CBT showed a minimal additional effect. Importantly, drop-out rates differed significantly for the two conditions (SC: 48.2%; RCBT: 26.7%). SC was effective in the treatment of panic disorder in terms of symptom reduction and remission rate, but dropout rates were twice as high as those seen in RCBT, with the second phase of SC not substantially improving treatment response. However, SC required significantly less therapist contact time compared to RCBT, and more research is needed to explore predictors of success for guided self-help interventions to allow treatment intensity to be tailored to patients' needs and preferences.

## Introduction

Cognitive Behavioural Therapy (CBT) and/or pharmacotherapy for panic disorder with or without agoraphobia (PD(A) are the treatments of choice according to international guidelines (e.g. [1,2]). These guidelines also advocate the use of stepped care (SC), i.e. to initially provide patients with the least intensive and thus least expensive treatment, to make treatment more widely accessible and affordable. In general, the model is simple, in that patients start with a low-intensity treatment (e.g. psycho-education, e-health, blended treatment), with the treatment being stepped up to a more intensive intervention (e.g. weekly face-to-face sessions, day-treatment) for those patients that do not respond (sufficiently) to the first phase of treatment to obtain the same treatment response after the second phase. The assumption is that some patients will not require the second stage of treatment. However, it is unclear what the effects are of stepped up treatment if all interventions are based on the same treatment rationale. It could be possible that the consecutive second step in the same treatment rationale is very effective because patients already are familiar with the treatment model and had a “pretreatment module”. On the other hand, it could also be possible that the consecutive next step is not effective because the first step did not work well and the treatment module lost its credibility when the same components are used again, even when given in a face-to-face treatment context.

Even though SC models are already implemented on a large scale, evidence for their effectiveness is still limited [3]. Three of the few available studies that have evaluated SC, compared the intervention with treatment as usual (TAU) as delivered in primary or collaborative care settings [4–6]. Although two of these studies report better effects for SC [4,6], they did not mention whether TAU consisted of- or was comparable to -an evidence-based treatment. To date, two studies [7,8] directly compared SC to evidence-based treatments for anxiety disorders. In one study, a low-intensity CBT for obsessive compulsive disorder was followed by face-to-face CBT if needed to guideline CBT was compared and one study. There were no differences in clinical outcomes for the two conditions, but the SC approach significantly reduced treatment costs [7]. The other, more recent study [8] also observed no differences in treatment effects between a low-intensity CBT followed by regular CBT if additionally needed for PD(A) or social anxiety disorder vs. regular CBT, albeit that dropout in the SC condition was significantly larger.

Although there are substantial advantages (such as making therapy as short as possible, as affordable as possible, and as acceptable as possible) to SC, the approach also appears to have certain drawbacks and issues in terms of higher rates of dropout and demoralisation [9], while the effect of intensifying the treatment based on the same rationale is also unknown [10,11].

The present study investigates the efficacy of SC targeting patients with PD(A) by directly comparing a low-intensity self-help CBT followed by regular CBT in case of non-response to prevailing international guideline treatment (RCBT or Treatment as Usual: TAU).

The hypotheses were that: 1) guided self-help is an effective first step in the SC treatment of panic disorder, compared with RCBT. We expected to find no difference in remission rates between groups at the end of both treatments; 2) we expected that in the second step of the stepped care intervention (which was manualized CBT), panic disorder symptoms would decrease faster as compared to the first step of SC because of familiarity with the therapeutic paradigm and procedure of CBT.

## Materials and methods

### Study design

Four specialised Dutch mental health-care institutions took part in this study: (1) Overwaal, Centre of expertise for Anxiety Disorders, OCD, and PTSD (Nijmegen/Lent), 2) GGZinGeest (Amsterdam), 3) HSK (Rosmalen, Nijmegen), and 4) Hendriks & Roosenboom (Arnhem). All participating practitioners were trained in SC by an expert senior clinician. The institutes participated in our 23-week Randomised Controlled Trial (RCT) with a follow-up I after one month and a follow-up II after six months. Patients were randomly assigned to one of two conditions: stepped care (SC) or regular CBT (RCBT). Primary measurements were taken at baseline and at every session. Secondary measurements were taken at baseline and every five weeks. Patients who were considered remitted, could stop treatment at any time. The trial was approved by an independent medical ethics committee (CMO Arnhem-Nijmegen, The Netherlands) and was conducted between March 2009 and July 2012. All patients gave their written, informed consent before study onset. The trial was retrospectively registered at ISRCTN36376369.

### Participants

All participants met the criteria for a primary diagnosis of PD(A) as defined by DSM-IV and were 18 years of age or older. Also, they had to have a minimum score of 10 on the Panic Agoraphobia Scale (PAS) [12]. The patients receiving SC who were taking medication for their PD(A) agreed to maintain on their current dose for the duration of the study, with the dose having been stable for six weeks before study entry. Exclusion criteria were having a: psychotic disorder, severe mood disorder, suicide risk, or substance dependence, all assessed with the Dutch version of the Mini International Neuropsychiatric Interview (MINI) [13] for DSM-IV. The MINI was conducted by experienced psychiatrists and psychologists, trained and qualified in assessing DSM disorders using the MINI. Additional exclusion criteria were: receiving ongoing treatment for PD(A) or a poor understanding of the Dutch language. At the start of treatment, 25.6% of the patients receiving SC used antidepressant medication (SSRIs), 22% benzodiazepines, and 6.1% a combination of both. In the RCBT condition this was 11.4%, 31.8%, and 0%, respectively. There were no significant differences in use of medication between conditions.

### Procedure

Patients were referred by their general practitioner and screened at the participating institutes after which patients with a primary PD(A) diagnosis were asked to join the study. After further screening on in- and exclusion criteria and (after a further week) a written informed consent had been obtained, patients completed the first assessment, immediately following by randomization. When patients were assigned to SC, they received the SC-Step 1 workbook. In each treatment condition, the first session was scheduled within two weeks after the baseline assessment. Because a score of <8 on the PAS indicates panic disorder remission, patients were considered as non-remitted when they still had a score of ≥ 8 on the PAS [12]. Patients in remission were free to stop their treatment phase at any point. When patients in SC were considered as remitted in Step 1, they could continue with Step 2, but they were no longer included in the study protocol. Interventions are described below in more detail.

### Randomisation

Patients were randomised to either SC or RCBT before study onset by an independent researcher. The allocation sequence was computer generated for each location separately and in fixed blocks of five. Based on Gould and Clum [14], we expected that Step 1 would be sufficient for at least 50% of the patients who received Step 1 of SC. Expecting a comparable number of patients to benefit from Step 2, we opted for a 2:1 ratio for the SC and RCBT conditions, in this way a comparable n was guaranteed to compare SC Step 2 with RCBT.

### Interventions

CBT interventions in all conditions were mainly based on the Panic Control Treatment manual by Craske & Barlow [15] which has proven to be effective in the treatment of PD(A) [16] and also has been proven to be effective in the Dutch population [17]. A comprehensive written manual was used to guide the patients in the SC condition. To ensure fidelity and adherence to protocol, therapists had been trained in SC and RCBT by an expert senior clinician (MK) and received regular weekly supervision The instructions to the therapists in the RCBT condition were to deliver CBT according to the prevailing international guidelines, with the possibility to augment CBT with psychotropic medication. Adherence to the treatment protocol was enforced by using checklists which were discussed at weekly supervision sessions. Adherence to protocol was 90%.

#### Stepped-care condition

The first step of SC comprised guided self-help. When patients were non-remitted (PAS score ≥ 8) they were advised to continue with Step 2, which consisted of manualized CBT.

*Step 1 Guided self-help*. Patients received a self-help workbook that was specifically designed for the present study in accordance with guideline CBT manuals for PD(A). The workbook contained psychoeducation, cognitive therapy, (interoceptive) exposure, and homework assignments. Every two weeks patients also had a meeting with their therapist. Each meeting took 30 minutes. Patients received the self-help manual two weeks before the first meeting with the therapist. The manual was divided in 5 subsections and patients were supposed to read and perform the homework assignments before meeting the therapist. Elements in section 1 consisted of psychoeducation, the treatment rationale, and learning to register panic attacks (PA). Elements in section 2 consisted of registration of PA, using interoceptive exposure, and cognitive therapy. Elements in section 3,4, and 5 consisted of registration of PA, interoceptive exposure, cognitive therapy, and exposure in vivo. Section 5 also discussed relapse prevention. Step 1 took 10 weeks to complete with 5 face-to-face therapy sessions.

*Step 2 Manualized CBT*. Patients without symptom remission following self-help interventions were offered the second step of SC. This second step consisted of 12 45-minutes sessions of manualized face-to-face CBT for PD(A) delivered by a different therapist. The Step 2-treatment lasted a maximum of 15 weeks (540 minutes) and contained psychoeducation, cognitive therapy, (interoceptive) exposure, and homework assignments. In Step 2, session 1 comprised psycho-education, treatment rationale and registration of PA; session 2 and 3 comprised mainly interoceptive exposure; session 4–7 cognitive therapy and behaviour experiments; in session 8–12 exposure in vivo was added and in the last session relapse prevention was addressed.

#### TAU: RCBT

Treatment in the RCBT condition consisted of (on average) 45 minutes of weekly face-to-face CBT according to regular prevailing international guideline treatment [2]. Treatment comprised psycho-education, (interceptive) exposure (in vivo), cognitive therapy, and relapse prevention. If necessary, pharmacotherapy with antidepressants could be added in discussion with a psychiatrist.

### Therapists

Therapists were master's-level psychologists, and registered psychotherapists, clinical psychologists, and psychiatric nurses. All had received extensive training in CBT and had regular supervision. Of the total of 44 therapists (79.5% female) participating in the study, 75% had less than five years of clinical experience after receiving their master’s degree, 20.5% 5–10 years, and 4.5% over 10 years. All therapists were experienced in the treatment of anxiety disorders. Master’s-level psychologists had completed their internship in the treatment of anxiety disorders.

### Measurements

#### Primary outcome measures

The primary outcome measure was the Dutch version of the *Panic and Agoraphobia Scale (PAS)* [12], a structured interview consisting of 13 items to be rated on a 5-point scale. Scores on the PAS have been demonstrated to have good reliability and sensitivity to change. A score of ≥ 10 indicates the presence of a PD(A) diagnosis [12].

The second primary outcome measure was the Dutch version of the *Outcome Questionnaire-45 (OQ-45)* [18,19], a 45 item patient-self-rated inventory using 5-point scales with three subscales: Symptom Distress, Interpersonal Relationship, and Social Role. The Dutch version of the OQ-45 has demonstrated adequate reliability and sensitivity to change [19,20].

#### Secondary outcome measures

The secondary, self-reported outcome measures, all rated on 5-point scales, were: the Dutch versions of the *Agoraphobic Cognitions Questionnaire (ACQ)* [21], a 14-item measure assessing catastrophic thoughts; the *Mobility Inventory (MI)* [22], a 27-item questionnaire with its three subscales assessing agoraphobic behaviour when alone (MI-AAL), agoraphobic behaviour when accompanied (MI-AAC), and the number of panic attacks during the past week. In the present study, the mean scores of the MI-AAL and MI-AAC were used; and the 17-item *Body Sensations Questionnaire (BSQ)* [21], which two subscales measure the fear of body sensations and the frequency of body sensations. In the present study we used the fear of body sensations subscale only. The ACQ and the MI and their Dutch adaptations were found to have good test-retest reliability, high internal consistencies, and reasonably concurrent validity. The BSQ and its Dutch adaptation were found to be highly internally consistent and reliable [21,23].

The PAS ad the OQ were administered at baseline and during each session to prevent data loss in case of attrition [24]. Before the treatment sessions the PAS and the OQ were assessed by the therapist. The assessment took 10–15 minutes. The other measures were completed every five weeks, at a first follow-up (FU1) four weeks after the last session, and at a second follow-up (FU2) scheduled six months (25 weeks) after the last session. These assessments were performed by independent assessors trained in the various assessments and who were blinded to the treatment condition.

### Power considerations and sample size

**Fig 1:**
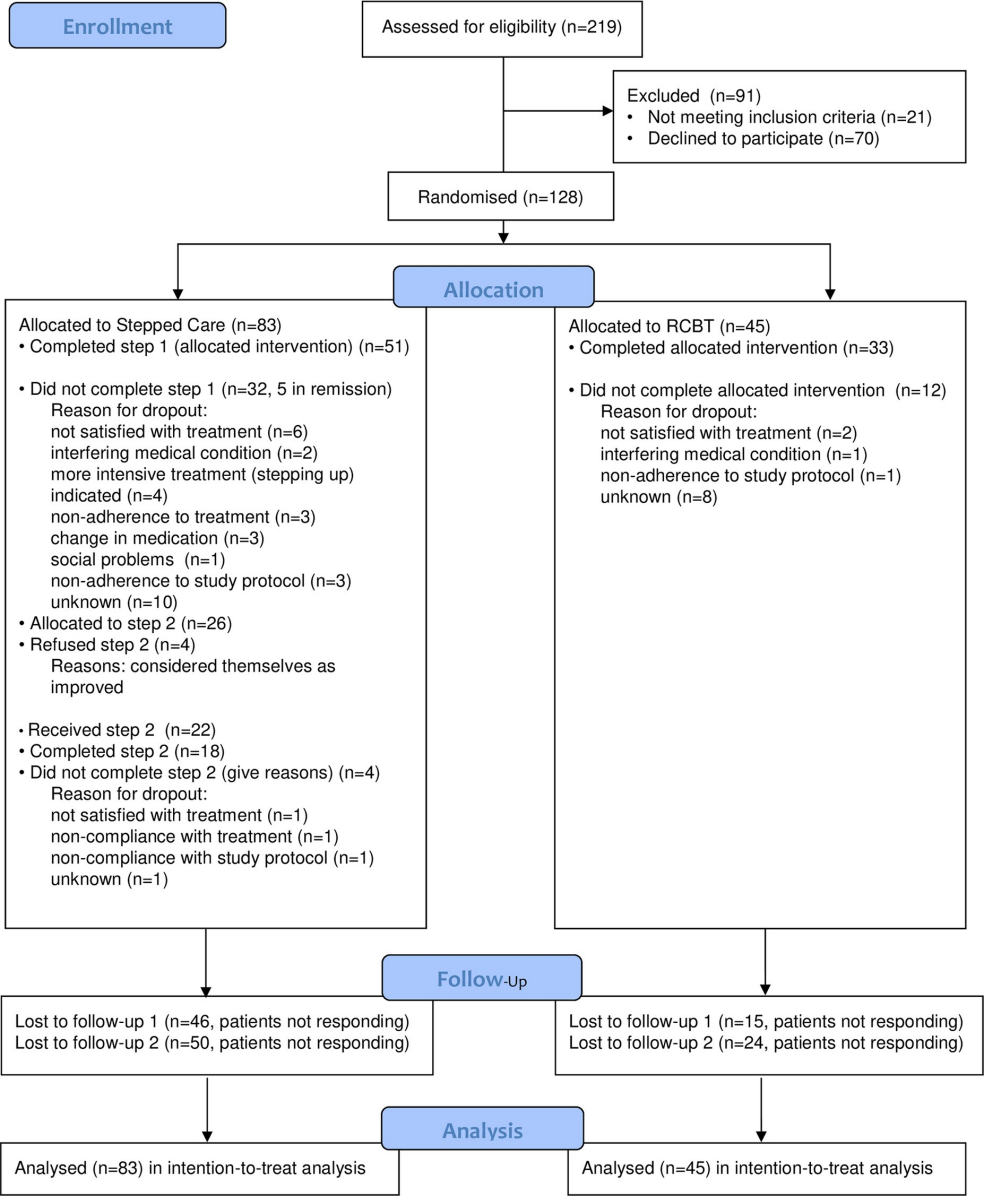
CONSORT Flow diagram.

The aim of our RCT was to compare the outcomes of the two separate treatment steps in patients receiving a maximum of 2 steps in SC and those receiving to those achieved with RCBT. Based on the previous research by Gould and Clum [14] we assumed that 50% of the SC patients would not have recovered after the first step (guided self-help) and would continue treatment. Being able to demonstrate at least moderate differences between the treatment arms with a power of .80 and alpha .05, we needed to include 40 participants in Step 2 [25]. Estimating that dropout would be around 20%, we aimed to include 150 patients in the study, with 100 patients being randomised to the SC and 50 to the RCBT-condition. The flow of the patients during the trial is depicted in Fig 1.

### Statistical analyses

Statistical analyses were conducted with IBM SPSS Statistical Package version 24.0 for Windows [25]. All statistical analyses were performed on an intention-to-treat basis. For dichotomised outcomes at posttreatment we used the Last Observation Carried Forward (LOCF) procedure and for the analyses with continuous measures all available data were analysed using the MIXED procedure.

We first computed the percentages of remitted and unremitted patients to compare outcomes (remission) per treatment condition. Patients who dropped out of the treatment could be remitted or unremitted according to the last score on the PAS. Differences in the percentages of remitted patients were analysed using Pearson Chi-Square tests.

Next, we compared the effects of the stepped-up intervention on the main outcome measures (PAS and OQ) for SC and RCBT by specifying a two-piecewise Random Coefficient Model [26] in the MIXED procedure of SPSS to model the trends over time in both conditions for both measures. The first piece of the model included Step 1 (SC group) and the first ten weeks of RCBT. The second piece included Step 2 (SC group) and all weeks following week 10 of RCBT. For the repeated effects, a diagonal covariance structure was specified.

The difference in mean scores between the groups at week 10 (end of Step 1) and week 25 (end point) were tested with the Estimated Marginal Means (EMMs) from the MIXED procedure. Additionally, custom contrasts were set up to compare trends between groups within the two pieces and to compare within-group trends between the two pieces. All available data from all subjects were used. The abovementioned analysis was repeated with the secondary outcome measures (ACQ, MI, and BSQ), albeit that now only EMMs were analysed.

All the statistical tests we performed, were 2 tailed tests at an alpha of 0.05.

## Results

### Participants flow

The initial screening procedure yielded 219 eligible subjects. Of these, 128 (58.45%) gave their informed consent, all of whom were included in the study and randomised to SC (n = 83) or RCBT (n = 45). Overwaal, centre of expertise for Anxiety Disorders, OCD, and PTSD (Nijmegen/Lent) included 68 patients, GGZinGeest (Amsterdam) included 20 patients, HSK (Rosmalen, Nijmegen) included 8 patients, and Hendriks & Roosenboom (Arnhem) included 32 patients. The flow of the patients from recruitment to the final ITT analysis and the reasons for dropout are presented in Fig 1. After 25 weeks, we had complete datasets for all 128 subjects (100%) included in the intention-to-treat sample. The per-protocol sample for SC included 43 patients (51.8%), of whom none refused the treatment condition they had been assigned to, while 40 (48.2%) dropped out. The per-protocol sample for RCBT included 33 subjects (73.3%), with none refusing the treatment they had been assigned to and 12 (26.7%) dropped out. The proportion of dropouts differed significantly for the two conditions (χ^2^(1) = 5.6, p < .05).

### Treatment delivery

The mean of the attended sessions in the SC condition was 5.9 (SD = 4.8; range 0–17) for ITT and 8.1 (SD = 4.9; range 2–17) for the completers, and in the RCBT condition 9.6 (SD = 5.6; range 0–20) and 11.3 (SD = 4.7; range 3–20), respectively. The difference in the number of attended sessions between the conditions was significant for both the ITT-group (*t*(126) = -3.87, *p* < .001) and the completers (*t*(77) = -2.92, *p* = .005), with more sessions having been attended in the RCBT condition.

In the SC condition no changes in medication were allowed and 78.3% of the patients did not change their medication use. However, 1.2% of the patients were found to have started an SSRI, 3.7% had decreased their use of SSRIs and 20.5% decreased their use of benzodiazepines, with 2.4% having increased the dose of benzodiazepines. The data of the medication use of 13.4% of the patients during the SC condition was missing.

In the RCBT condition 56.9% of the patients did not change their medication; 6.8% decreased their use of SSRIs, 20.5% decreased use of benzodiazepines, while 2.3% increased the use of benzodiazepines. No patients started using SSRIs during CBT. The data of the medication use of 13.5% of the patients during the SC condition was missing.

### Pretest characteristics

Table 1 presents patients’ demographics and characteristics. No significant differences emerged between the two conditions in any of the demographic data, in the primary and the secondary outcome measures at baseline.

**Table 1:** Patient demographics and symptom profiles.

	SC			RCBT		
	*N*	*M* or %	*SD*	*N*	*M* or %	*SD*
Age	83	39.8 (Median = 38)	12.1	45	35.5 (Median = 36)	11.4
Male	83	50.6%		45	51.1%	
Average or higher education	83	74.4%		45	73.3%	
Married/cohabiting	83	54.2%		45	57.8%	
Duration of disorder (yrs)	83	7.1 (Median = 3)	10.0	45	5.5 (Median = 2.25)	8.8
Comorbidity						
*Anxiety Disorders*		20.7%			25%	
Mood Disorder		19.5%			13.6%	
Somatoform Disorder		9.8%			6.8%	
Cluster C Personality Disorder	82	9.8%		42	4.5%	
*Use of Medication*	82			42		
Antidepressant		25.6%			11.4%	
Anxiolytic		22%			31.8%	
Combination antidepressant and anxiolytic		6.1%			0%	
Received prior treatment	83	68.7%		44	71.1%	

SC = Stepped Care; RCBT = regular cognitive behaviour therapy.

### Outcome

#### Patients in remission

*Stepped care*. In the SC condition 25 patients (30.1%) were remitted after Step 1. After Step 2, another seven patients (8.4%) were in remission. In total, of the 43 patients who completed treatment, 32 (74.4%) were remitted after both Step 1 and Step 2. Another five patients (6%) who were considered dropout in Step 1 were in remission. Of the 83 patients who started treatment, 37 (44.6%) were in remission.

*RCBT*. Of the 33 patients who completed RCBT, 21 patients were remitted (63.3%). Another three patients (6.7%) who had dropped out before the end of treatment were in remission. In total, 24 of the patients (53.3%) who started RCBT were in remission after treatment.

**Fig 2:**
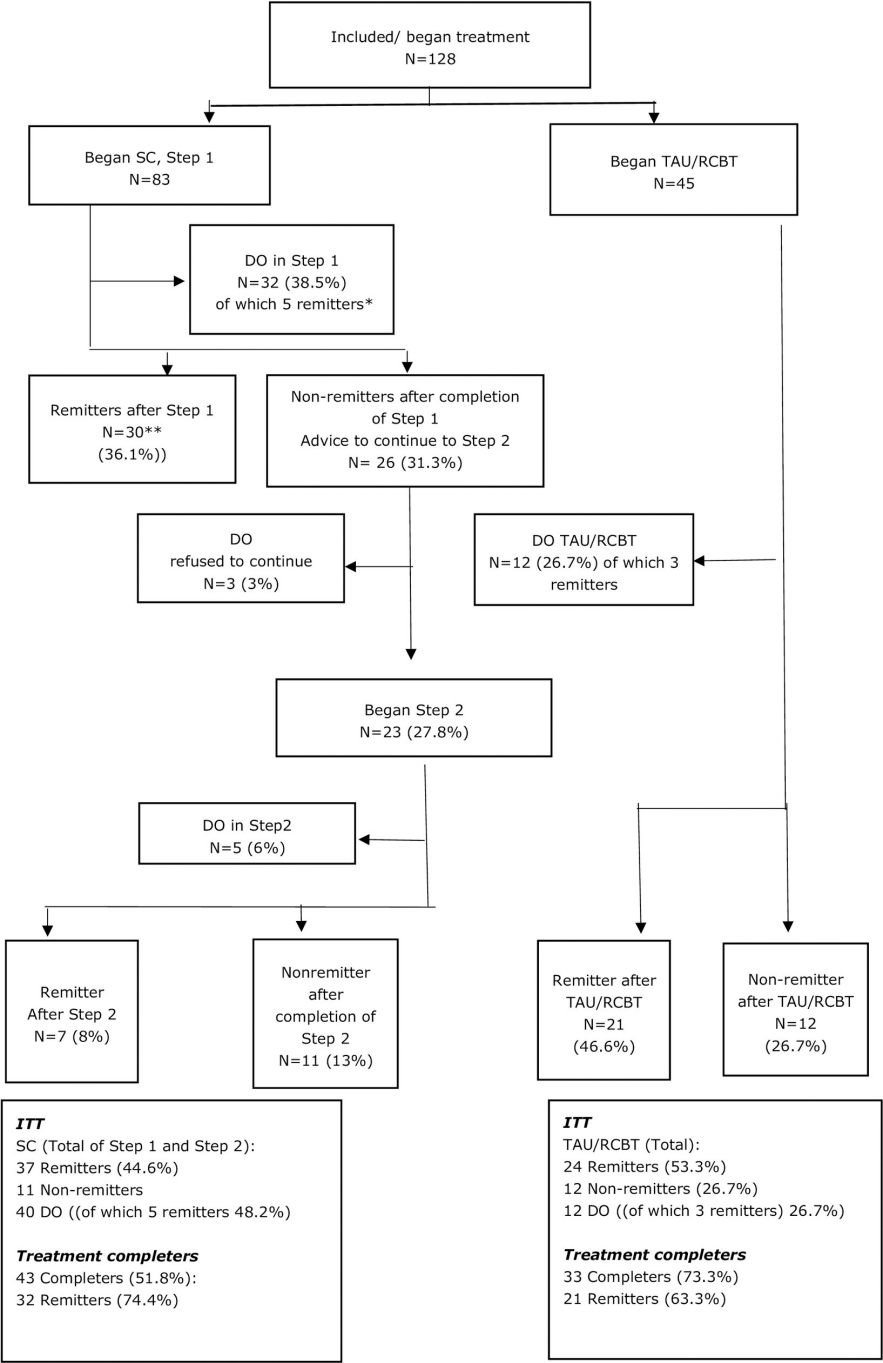
Flowchart of remission rates and dropout. SC = Stepped Care; RCBT = regular cognitive behaviour therapy; DO = dropout; ITT = Intention to Treat. *these patients remitted during the first step and did not want to complete the first step. **including the patients who remitted and did not complete the first step.

The percentages of remitted patients for the completers at posttreatment did not differ between the two conditions *(χ*^2^(1) = .546, *p* = .460). The percentages of remitted patients for completers at FU1 also did not differ significantly between the two treatment conditions (resp. *(χ*^2^(1) = .572, *p* = .449) but did differ significantly for completers at FU2 *(χ* ^2^(1) = .869, *p* = .027). Fig 2 depicts a flowchart of remission rates and dropout.

*The effects of Step 2 of SC versus the effects of RCBT*. Table 2 gives an overview of the EMMs and the p values of the PAS, OQ, ACQ, MI, and BSQ assessed at pretreatment, posttreatment, and the 4-week and 6-month Follow-Up (FU). There were no significant differences between the two treatment conditions for the primary and secondary outcome measures at all four time points, except for FU2 at six month for the PAS-score.

**Table 2:** F-test-statistics (two piece RCM) for differences between the two treatment conditions for the primary and secondary outcome measures at pre- and posttreatment, and after 4 weeks and six months.

		Stepped Care			RCBT			
		*N*	Estimated Means [95%CI]	*ES*	*N*	Estimated Means[95%CI]	*ES*	*p*
PAS	Pre-treatment	83	21.51 [19.64–23.37]		45	21.95 [19.45–24.44]		
	Post-treatment	47	10.96 [8.82–13.1]	1.53	34	10.04 [7.44–12.64]	1.66	.588
	Follow-up 4 weeks	36	10.23 [8.08–12.37]	1.63	29	8.68 [6.08–11.28]	1.85	.365
	Follow-up 6 months	32	7.11 [4.39–9.84]	2.08	23	2.91 [-0.26–6.09]	2.69	.049
OQ-45	Pre-treatment	83	68.61 [63.83–73.38]		45	64.64 [58.19–71.09]		
	Post-treatment	45	50.11 [44.3–55.92]	0.9	32	50.2 [42.85–57.54]	0.9	.986
	Follow-up 4 weeks	36	48.99 [43.02–54.96]	0.96	27	48.85 [41.37–56.34]	0.97	.978
	Follow-up 6 months	28	44.22 [36.51–51.92]	1.19	23	43.15 [34.12–52.19]	1.24	.859
ACQ	Pre-treatment	83	29.86 [27.99–31.73]		45	32.44 [29.92–34.96]		
	Post-treatment	45	24.41 [22.36–26.46]	0.77	29	25.53 [22.97–28.09]	0.61	.501
	Follow-up 4 weeks	36	23.98 [21.94–26.02]	0.83	27	25.03 [22.5–27.56]	0.68	.522
	Follow-up 6 months	29	22.16 [19.5–24.83]	1.08	23	22.94 [19.82–26.05]	0.97	.709
MI-AAC	Pre-treatment	83	2.02 [1.84–2.19]		45	1.87 [1.63–2.1]		
	Post-treatment	45	1.58 [1.41–1.74]	0.7	30	1.53 [1.32–1.74]	0.76	.758
	Follow-up 4 weeks	36	1.54 [1.38–1.71]	0.75	27	1.49 [1.29–1.7]	0.83	.691
	Follow-up 6 months	29	1.41 [1.21–1.6]	0.96	34	1.32 [1.09–1.56]	1.09	.589
MI-AAL	Pre-treatment	83	2.56 [2.35–2.76]		45	2.31 [2.03–2.59]		
	Post-treatment	45	1.88 [1.67–2.08]	0.87	30	1.84 [1.58–2.1]	0.92	.815
	Follow-up 4 weeks	36	1.83 [1.63–2.03]	0.92	27	1.77 [1.51–2.02]	1.01	.691
	Follow-up 6 months	29	1.64 [1.41–1.87]	1.17	23	1.46 [1.18–1.74]	1.39	.335
BSQ	Pre-treatment	82	90.87 [86.22–95.53]		45	93.08 [86.81–99.35]		
	Post-treatment	45	69.57 [64.22–74.91]	1.24	30	70.91 [64.4–77.43]	1.16	.752
	Follow-up 4 weeks	35	68.19 [62.91–73.47]	1.32	27	69.56 [63.17–75.96]	1.24	.743
	Follow-up 6 months	28	62.33 [55.66–68.99]	1.66	23	63.83 [56.14–71.52]	1.57	.769

SC = Stepped Care; RCBT = Regular cognitive behavioural therapy; PAS = Panic Agoraphobia Scale; OQ-45 = Outcome Questionnaire-45; ACQ = Agoraphobic Cognition Questionnaire; MI = Mobility Inventory; BSQ = Body Sensations Questionnaire. *ES*: Effect sizes were computed as the difference between the EMM and the EMM pre-treatment divided by square root of the model estimate of the variance of the measure at pre-treatment.

#### PAS and OQ

*Between conditions*. The slopes of improvement on the PAS for Step 1 of SC did not differ significantly from the slopes during the first 10 weeks of RCBT (*t*(83.05) = .47, *p* = .642). In Step 2 of SC, the PAS slopes did differ significantly from the further improvement achieved after the first 10 weeks of RCBT, in favour of RCBT (*t*(79.54) = 2.41; *p* = .018). This means that in the first 10 weeks of treatment the speed of improvement was comparable for the two treatment conditions but that after this period the patients receiving RCBT continued to improve significantly faster than the patients receiving Step 2 of SC. The posttreatment scores on the PAS and FU1 did not differ between conditions. At FU2, patients receiving RCBT had a significantly lower posttreatment score than patients receiving SC (*F*(81.75) = 3.99, *p* = .049).

The slopes of improvement on the OQ in Step 1 of SC did not differ significantly from the slopes for the first 10 weeks of RCBT (*t*(78.40) = -1.56, *p* = .124), nor did the slopes for Step 2 differ (*t*(65.84) = .363, *p* = .718), implying that the speed of improvement on the OQ did not differ between treatment stages or conditions, the posttreatment and FU scores also showing no difference.

*Within conditions*. Comparing the slopes of the two SC phases, we found the PAS improvement in the first step to be significantly faster than the improvement in de subsequent step (*t*(124.43) = -6.40, *p* < .001*)*. The improvement in the first 10 weeks of RCBT was also significantly faster than the improvement achieved after this period (*t*(82,88) = -5.10, *p* < 001). Accordingly, symptom reduction as assessed with the PAS was faster in the first treatment phase in both conditions. Similarly, the improvement on the OQ in the first SC phase was significantly faster than the gains in de second phase (*t*(120.87) = -6.12, *p* < .001), as were those recorded in the first 10 weeks of RCBT compared to the symptom reduction after 10 weeks (*t*(83.09) = -3.00, *p* = .004).

#### ACQ, MI, and BSQ

See Table 2 for the EMMs of the ACQ, MI, and BSQ. There were no significant differences in posttreatment and follow-up outcomes for the two conditions.

## Discussion

### Main findings

The results of our RCT comparing stepped care (SC) and regular CBT (RCBT) for panic disorder showed no significant differences in the number of remitted patients between both conditions. The outcomes on the primary and secondary measures were similar at all assessments, except for the PAS at FU2, where patients in RCBT had lower scores. In contrast, the percentages of remitted patients who completed treatment differed significantly in favour of SC. Overall, the effect sizes within the conditions were medium to large. It needs to be noted, however, that the response rate for FU2 was low (33% in SC, compared to 47% in RCBT). This finding should thus be interpreted with caution. Although the patients having received SC had attended significantly fewer sessions, the number of patients dropping out of treatment prematurely was twice as high as the number dropping out of RCBT.

Symptom reduction assessed with the PAS and OQ-45 was faster in the first 10 weeks (coinciding with SC-Step 1) in both treatments, with further gains after SC-Step 2, being lower than the gains achieved in the later stages of RCBT. Accordingly, in both conditions the majority of responders benefited most from the first part of the intervention. The assumption that patients need less therapy after a pre-treatment module of a therapy in the same treatment rationale was thus not confirmed. Our results suggest that the present SC model helps lower costs in terms of reducing therapist contact time without negatively affecting treatment outcomes and this could make treatment more accessible.

However, there are two main considerations. (1) Unsuccessful patients in need of the second step of treatment in the SC condition did not respond as well as the patients in the second stage of RCBT did. Importantly, stepping up the treatment with face-to-face CBT after the initial self-guided CBT did not boost recovery. Apparently, patients with PD(A) are less likely to profit from an intensified version of the same treatment. (2) Dropout was twice as high in the SC condition. Some of the patients who did not (sufficiently) remit in Step 1 did not continue their treatment, with the potential risk of undertreatment, demoralisation, a chronic course of their PD(A), and negative social consequences.

### Limitations

The first limitation of the present study that has to be mentioned is that the amount of inclusions according to the power calculations has not been met. The second limitation is the large amount of patients leaving Step 1 prematurely. Therefore, although perhaps indicative of the problems posed by SC approaches, the results of this study have to be interpret with caution.

Another possible limitation is the guided self-help module we used concerned a pen-and-paper version, specifically designed for the present study rather than an online version, which would now possibly be preferred as there are nowadays many versions of e-health treatment modules available. The 48.2% dropout rate (41% without the remitted patients who dropped out earlier during the trial) in step 1 of our SC protocol is problematic. Previous SC studies reported drop-out rates ranging from 10% to 55.3% [4–8]. It is likely that guided self-help approach is not tolerated or accepted by large numbers of patients. We also need to mention the large number of therapists and novice therapists participating in the study, which may have biased our treatment outcomes, although all therapists in the participating anxiety disorder centres were experienced in treating panic disorder and had received extensive training and supervision. However, differences in therapist competence was found to be associated with (poorer) treatment outcome in some studies [8] but not in others [27]. We did not have sufficient numbers to analyse whether therapist competence played a role in the current study.

Lastly, we did not evaluate the cost-effectiveness of the two treatment conditions but used estimated therapist contact time as an indication of cost per treatment. Besides this, in the original research protocol we planned to evaluate pretreatment predictors, which would be helpful in treatment allocation (self-help treatment versus RCBT). Because of the large proportion of dropouts in Step 1 of the SC, the analyses would be underpowered and not comparable to the RCBT-condition.

### Comparison with the literature

Although there is ample research about the effectiveness of e-health or (guided) self-help programmes, only few studies examined the effects of consecutive psychological treatment strategies in a stepped-care approach to panic disorder.

The findings of the present study are consistent with previous findings on the effectiveness of SC for anxiety disorders [4,6–8]. Comparing the completer outcomes of two stages of SC with regular CBT and with 74.4% of the patients having received SC being in remission after the second step of manualized treatment, we can conclude that SC can be effective in the treatment of panic disorder. Of the patients having completed RCBT, 63.6% was in remission. ITT remission rates were 44.6% and 53.2% for SC and RCBT, respectively, which is consistent with Nordgreen et al. [8] who reported a 40% recovery rate for SC and a 43% recovery rate for regular face-to-face treatment.

Dropout from the first step of SC was higher (38.5%) than the dropout reported for other low-intensity or guided self-help treatments [4,6,7,28], where rates varied between 5 and 27%. However, our rate was comparable to the rate Nordgreen [8] recorded. The lower dropout in previous trials might be explained by the fact that in most of these studies the first phase of treatment was delivered in primary care settings [4–6]. In Nordgreen's [8] and our study, Step 1 was delivered in specialised units, where patients might have expected some form of face-to-face treatment rather than a guided self-help programme more common in primary care settings.

Another risk in SC is that almost half of patients who did not recover sufficiently, subsequently rejected the next stage of the programme and thus remained undertreated. Delgadillo et al. [29] found that in low-intensity treatments reliable improvement in the first three sessions predicted treatment outcome. Also Schibbey et al. [30] found that for an internet based CBT for panic disorder, the results on the disorder specific measure predicted treatment response at week 4 of treatment. Tiemens et al., [31] found that low frequency sessions at the initial start of treatment predicted a chronic course in the treatment of anxiety disorders in a natural treatment setting. This means that stepping up to a more intensive treatment in time by monitoring carefully to predict the course of the treatment and a session frequency of one or two times a week may prevent dropout or delay of remission. If improvement is delayed in the first phase of the intervention, patients might get demoralised and drop out of treatment altogether, potentially increasing the individual, medical, and social burden. This should be further explored in future research. In general, most patients drop out in the first five sessions [32], while after 10 sessions further treatment response is moderate, emphasising the importance of early and continued progress monitoring and targeted and more patient-specific interventions when necessary. Also in view of the recent findings of Ali and colleagues [33] who emphasise the risk of relapse after low-intensity treatments, we need to be aware that SC programmes are no simple solutions to reducing waiting lists and costs.

Little is known about the acceptability of low intensity programmes or stepping up in the same treatment module. The shared decision-making between patient and therapist is of importance to manage expectations and perhaps prevent dropping out in the first sessions [34]. Also, there is need for further research into the effects of treatment when a second step is a more intensified phase of the first step [35].

### Implications

The main findings of the present study confirm that stepped care can be effective in the treatment of panic disorder, improving treatment accessibility while containing costs. However, the study also demonstrates the need for careful implementation of SC programmes in specialised treatment centres given the higher risk of patients dropping out, with the associated risk of creating a group of demoralised, undertreated, and thus potentially chronic patients. SC programmes may be more suitable for primary care centres and conducted by trained mental health nurses. Moreover, patients who do not respond to low-intensity CBT (guided self-help) do not necessarily respond to the next phase of treatment (face-to-face CBT). Before SC models are implemented on a wider scale, additional research is required to explore predictors of success for guided self-help.
